# QTL mapping of drought-related traits in the hybrids of *Populus deltoides* ‘Danhong’×*Populus simonii* ‘Tongliao1’

**DOI:** 10.1186/s12870-022-03613-w

**Published:** 2022-05-11

**Authors:** Changjian Du, Pei Sun, Xingqi Cheng, Lei Zhang, Lijuan Wang, Jianjun Hu

**Affiliations:** 1 State Key Laboratory of Tree Genetics and Breeding, Key Laboratory of Tree Breeding and Cultivation of National Forestry and Grassland Administration, Research Institute of Forestry, Chinese Academy of Forestry, Beijing, 100091 China; 2 Co-Innovation Center for Sustainable Forestry in Southern China, Nanjing Forestry University, Nanjing, 210037 Jiangsu China; 3 Institute of Forestry and Pomology, Beijing Academy of Agriculture and Forestry Sciences, Beijing, 100093 China

**Keywords:** Drought stress, QTL mapping, Crossbreeding, *Populus*, Coexpression network, Drought-related traits

## Abstract

**Background:**

Poplar trees provide a large amount of wood material, but many parts of the world are arid or semi-arid areas because of insufficient annual precipitation, which seriously affects the growth of poplar trees. *Populus simonii* ‘Tongliao1’ shows strong tolerance to stress environments, and *Populus deltoides* ‘Danhong’ shows a stronger growth rate in a suitable environment. To identify drought tolerance-related QTLs and genes, an F_1_ population derived from the cross between the ‘Danhong’ and ‘Tongliao 1’ *Populus* was assessed under drought stress.

**Results:**

We measured drought-related traits such as the relative height growth, relative diameter growth, leaf senescence number, specific leaf area, and leaf relative water content in the population under control and drought environments. The results showed that drought stress reduced the plant height relative growth, ground diameter relative growth, specific leaf area and leaf relative water content and increased the number of leaf drops. A total of 208 QTLs were identified by QTL mapping analysis, and they consisted of 92, 63 and 53 QTLs under control, drought stress treatment and drought index conditions, respectively. A molecular identification marker for drought tolerance, np2841, which was associated with a QTL (qDLRWC-LG10-1) for relative leaf water content, was initially developed. We mined 187 candidate genes for QTL regions of five traits under a drought environment. The reference genome annotation for *Populus trichocarpa* and a homologous gene analysis of *Arabidopsis thaliana* identified two candidate genes, *Potri.003G171300* and *Potri.012G123900*, with significant functions in response to drought stress. We identified five key regulatory genes (*Potri.006G273500*, *Potri.007G111500*, *Potri.007G111600*, *Potri.007G111700*, and *Potri.007G111800*) related to drought tolerance through the poplar coexpression network.

**Conclusion:**

In this study, our results indicate that the QTLs can effectively enhance the drought tolerance of poplar. It is a step closer towards unravelling the genetic basis of poplar drought tolerance-related traits, and to providing validated candidate genes and molecular markers for future genetic improvement.

**Supplementary Information:**

The online version contains supplementary material available at 10.1186/s12870-022-03613-w.

## Background

Poplar (*Populus*) is an important industrial wood raw material and has characteristic fast growth, strong adaptability, and high yield; it has been cultivated in extensive areas in China and all over the world [1, 2]. Because of the impact of rainfall in different distribution areas, poplar growth is easily inhibited by drought stress, threatening its growth and development, yield, quality, and even causing large dead areas [3, 4]. Research on the drought tolerance of poplars is primarily performed by individuals, and few studies have been dedicated to constructing hybrid populations and selecting drought tolerance genes [5–7]. Poplar breeding target traits are mostly quantitative characteristics that regulated by multiple genes with different genetic effects, such as additive, codominant and epistatic effects. Quantitative trait locus (QTL) mapping is a formal genetic research method for resolving the genetic basis of poplar drought-related traits, exploring candidate genes and developing functional molecular markers [8–10].

The drought tolerance trait of poplar is a quantitative characteristic, and its genetic mechanism is extremely complex, and is involved in many biological metabolic pathways [11–14]. Conventional breeding involves a long cycle time, poor foresight, low selection efficiency, and an inability to identify multiple genes controlling drought tolerance. With the rapid development of high-throughput sequencing technology, molecular breeding has played an important role in the study of quantitative characteristics. Research on QTL mapping in forest trees started after that of crops, but greater progress has been made [15]. The QTL mapping work on poplar primarily focused on important economic traits such as growth and wood properties [16–18]. At present, there are few studies on the deep analysis of drought tolerance-related quantitative characteristics in poplar, and it is an urgent problem to solve [5, 19]. Poplar was the first to be sequenced among woody species, and its genome information is relatively complete. After a genetic map and QTL mapping of the target trait are constructed, the markers in the target QTL interval can be directly mapped to mine candidate genes [20].

Techniques for mining plant drought-related candidate genes include genetic mapping, bulked segregant analysis (BSA), genome-wide association study (GWAS), RNA sequencing, etc. [19, 21–24]. To analyse the genetic basis of drought-related traits in poplars from a population genetics perspective, we used high-density genetic mapping to mine the QTLs for target biological traits and to screen candidate genes [25]. QTL mapping can help us identify multiple regulatory genes for a target trait, making up for the shortcomings of single gene analysis studies. In addition, the determination of QTL positions in the genome can help researchers to find molecular markers associated with these positions, providing a reference for mining candidate genes with master-effect QTL regions and laying the foundation for fully understanding the molecular regulatory mechanisms underlying target biological traits [26]. Tschaplinski et al. [17] established field test stands in the Boardman and Clatskanie areas with differential climatic conditions and irrigated with different water during the growing season. Using *P. trichocarpa* ×*P. deltoides* F_2_ populations as material, 12 QTLs were identified for infiltration potential traits with a range of 5.5-19.1% variation in explained phenotypic variation [27]. Viger et al. established a field trial stand of *P. trichocarpa* ×*P. deltoides* F_2_ populations using drought stress treatments in northern Italy and south-eastern England with differential rainfall and identified 10 QTLs associated with isotope C^13^ and two QTLs associated with stomatal conductance, three recombination hotspots and 23 candidate genes in response to drought [28]. In short, the comparative analysis of the genetic regulation mechanism underlying drought tolerance in different genetic background populations must be further studied.

Hybrid breeding is the most widely used method for breeding new poplar varieties. By selecting individuals with superior genotypes from different seed sources and lines as parents, we can create crossbreeding populations with rich genetic variation to help us select superior new varieties [29]. *P. deltoides* (*Aigeiros*) is fast-growing and disease tolerant, with good stem shape and high economic value. Over 90% of the poplar species currently cultivated in the world originate from the *Aigeiros* species, but most of them are less resistant to environmental stress, such as drought and salt [30]. *P. simonii* (*Tacamahaca*) is a native tree species of China, that is cold resistant, drought, tolerant, and alkaline resistant, has a well-developed root system and strong wind resistance and is an important protective and timber forest species in northeast and northwest China [9, 31]. Under a natural environment, *Aigeiros* and *Tacamahaca* are prone to producing natural hybrids, and have obvious heterosis. The two species differ significantly in many traits, but there is no reproductive isolation in distant hybrids. Therefore, the cross can yield the ideal segregating population, which can breed new poplar varieties with good resistance and wide adaptability.

‘Danhong’ (*P. deltoides*) has excellent characteristics such as rapid growth and straight stem shape, but it requires good water and fertilizer conditions and is drought-sensitive [5]. ‘Tongliao 1’ (*P. simonii*) has excellent resistance to cold temperatures and weak alkaline soil, but its growth is slow [5]. In this study, drought tests were conducted on F_1_ populations to determine seedling drought-related traits and QTL mapping and mine candidate genes to lay the foundation for future breeding using molecular marker-assisted breeding and genetic improvement of drought tolerance.

## Results

### Phenotypic trait analysis

**Fig. 1:**
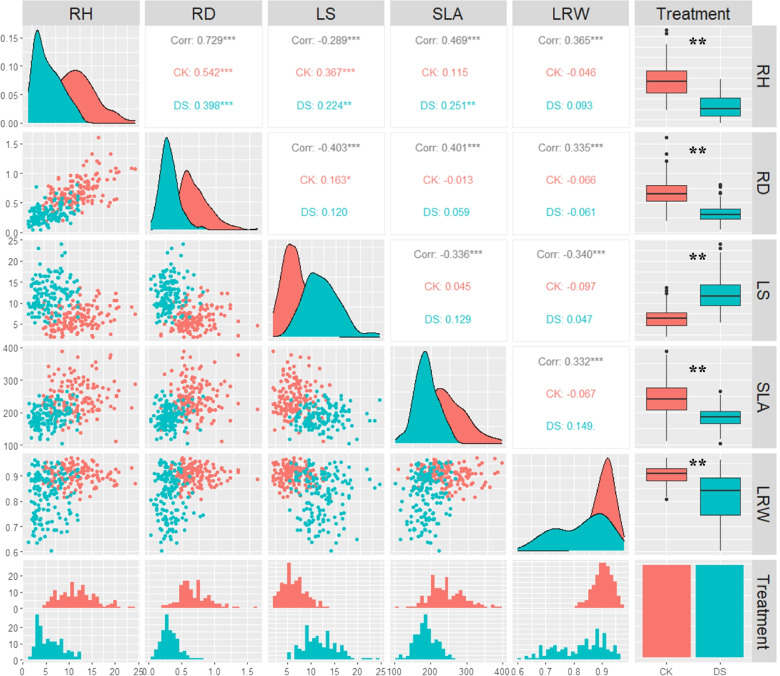
Phenotypic analysis of F_1_ populations under drought and control environments. CK: control environment, red; DS: drought environment, light blue; frequency distribution histogram (on the below); comparative analysis of phenotype data under drought and control environments by boxplots (on the right); density dissolution curve (on the diagonal); correlation analysis (above diagonal), CK was control, DS was drought condition, corr was integrated control and drought treatment data; and scatter plot for correlation analysis (below the diagonal); Asterisks show the different degrees of significant positive or negative correlation, respectively. *, **, and *** indicate significant difference at the *P* <0.01, 0.05, and 0.001 levels, respectively

To explore the inner relationship among drought-related traits, a correlation analysis (Pearson correlation) was performed (Fig. 1, above and below the diagonal). There was a significant difference in the correlation between the traits under drought and the control environment. Integrating the phenotypic data analysis on drought treatment and control revealed that the relative diameter growth (RD) and relative height growth (RH) became highly positively correlated, *R*^2^ = 0.729, *P* < 0.001; there was a positive correlation between the relative height growth (RH) and specific leaf area (SLA), *R*^2^ = 0.469, *P* < 0.001; there was a positive correlation between the relative diameter growth (RD) and specific leaf area (SLA), *R*^2^ = 0.401, *P* < 0.001; there was a negative correlation between the leaf senescence number (LS) and relative height growth (RH), *R*^2^ = -0.403, *P* < 0.001; and there was a weak negative correlation between the leaf relative water content (LRW) and the other four traits. The histogram showed that the phenotypic data under drought and the control environment followed a normal distribution (Fig. 1, below). The comparison between the control and drought stress phenotypic data showed that the RH, RD, LS, SLA, and LRW experienced obvious changes through the density dissolution curve (Fig. 1, diagonal). The box diagram indicates that the RH, RD, SLA, and LRW decreased after drought treatment, and the LS increased, *P* < 0.01 (Fig. 1, right). In summary, the above analysis showed that drought treatment had a significant effect on the F_1_ populations, and the RH, RD, LS, SLA, and LRW traits significantly responded to drought stress and could be used as traits for drought tolerance QTL mapping.

**Fig. 2:**
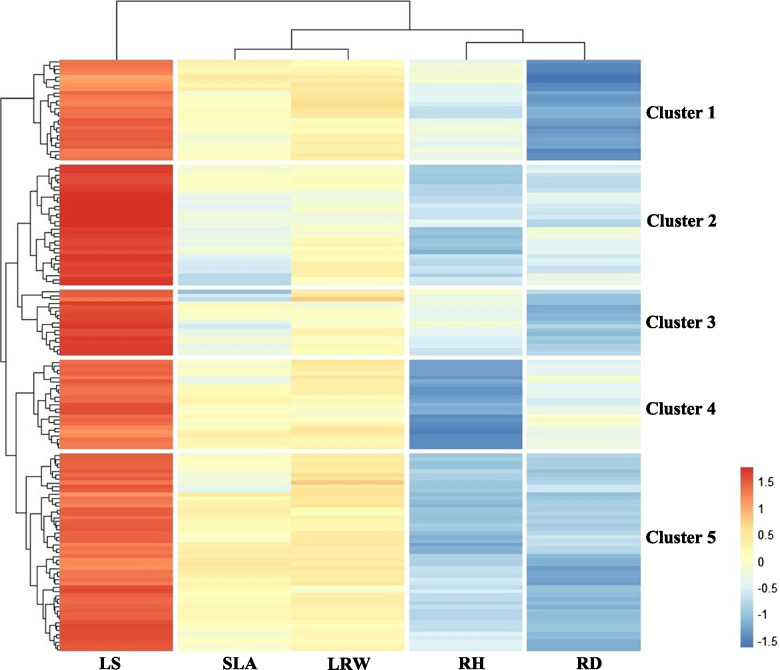
Heat map of the cluster analysis on the drought tolerance index

We calculated the means and heritability for the traits in the parental and F_1_ populations (Table 1). The difference analysis between parents showed that the RH of ‘Tongliao 1’ was significantly higher than that of ‘Danhong’ poplar in both the control and treatment groups; The LS of ‘Danhong’ was significantly higher than that of ‘Tongliao 1’ in the drought group; and the SLA of ‘Danhong’ was significantly higher than that of ‘Tongliao 1’ in the control group. The coefficient of variation of the F_1_ population traits varied between 0.03-0.47, showing that the populations had abundant genetic variation in drought tolerance traits. We calculated the drought index for five traits, RH, RD, LS, SLA, and LRW, which can respond to the drought response of each individual. The drought indices of the five traits were analysed by clustering and plotting the heat map (Fig. 2). For the cluster analysis, we divided the F_1_ populations into five clusters. The RH indices of Cluster1 and Cluster3 were higher, showing that their drought tolerance was stronger.

**Table 1:** Statistical analysis of phenotypic data from parental and F_1_ populations

Phenotypic traits	Treatments	***P. deltoides*** ‘Danhong’	***P. simonii*** ‘Tongliao1’	F_**1**_ populations (Average ± SD or SE)	Variation coefficient	Heritability
**Relative height growth (RH)**	Control	5.54	8.97^a^	11.90 ± 4.02	0.34	0.82
	Drought	2.83	5.8^a^	5.85 ± 2.75	0.47	0.79
	Drought index	0.52	0.65	0.51 ± 0.15	0.29	–
**Relative diameter growth (RD)**	Control	0.19	0.2	0.69 ± 0.22	0.32	0.34
	Drought	0.15	0.13	0.32 ± 0.13	0.41	0.23
	Drought index	0.75	0.71	0.49 ± 0.14	0.29	–
**Leaf senescence number (LS)**	Control	3	2	6.48 ± 2.56	0.4	0.72
	Drought	9	5^a^	12.11 ± 3.59	0.3	0.7
	Drought index	3.56	2.39	2.15 ± 0.58	0.27	–
**Specific leaf area (SLA)**	Control	213.64	145.84^a^	245.42 ± 50.65	0.21	0.74
	Drought	163.56	117.15	187.33 ± 30.21	0.16	0.71
	Drought index	0.76	0.8	0.79 ± 0.11	0.14	–
**Leaf relative water content (LRW)**	Control	0.95	0.73	0.90 ± 0.03	0.03	0.19
	Drought	0.93	0.75	0.74 ± 0.09	0.12	0.16
	Drought index	0.77	0.81	0.82 ± 0.09	0.11	–

^a^significant difference between *P. deltoides* ‘Danhong’ and *P. simonii* ‘Tongliao1’ at *P* < 0.05

### Principal component analysis

**Fig. 3:**
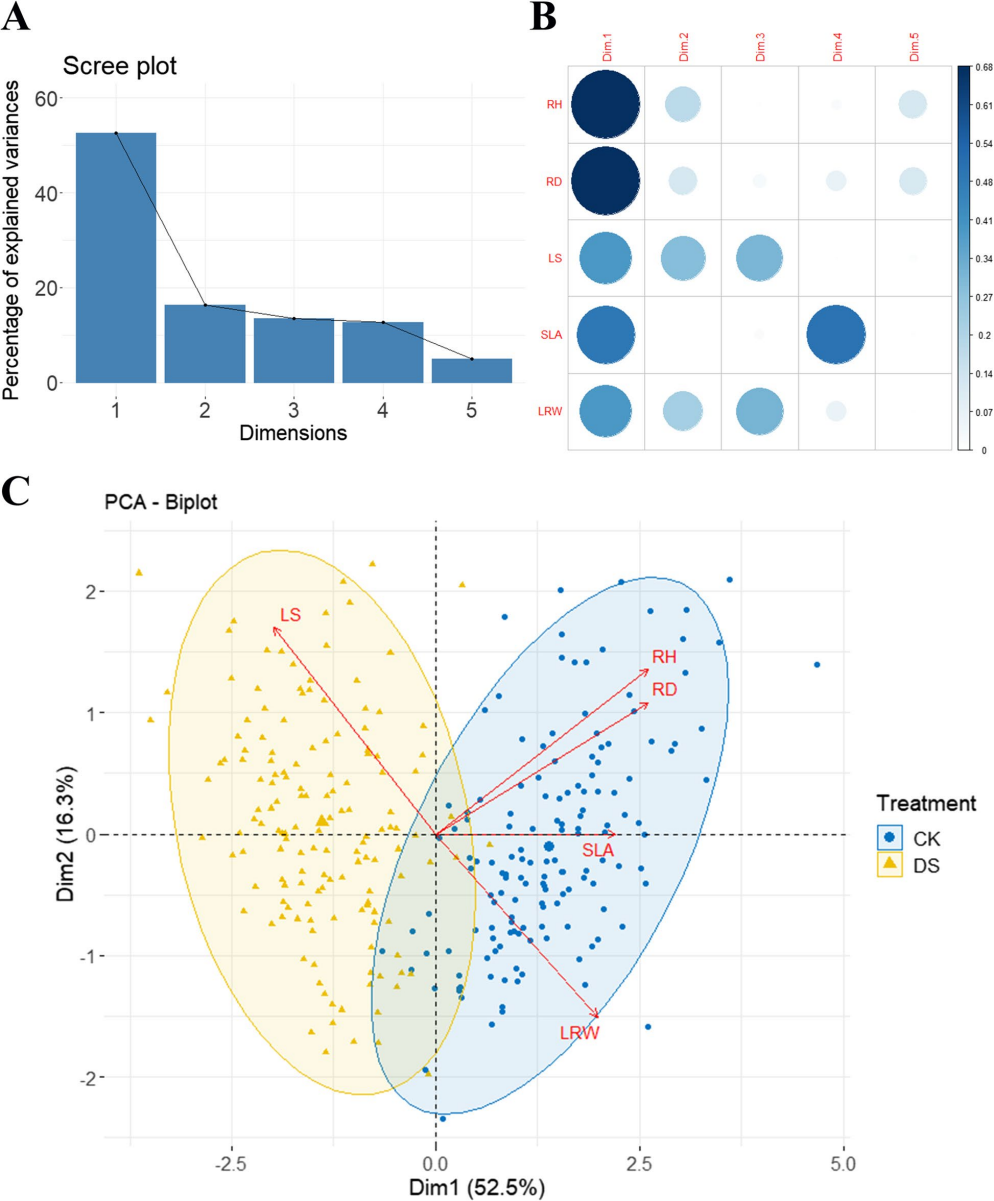
Principal component analysis of population phenotypes under control and drought environments. **A** Scree plot, the variance contribution rate of each principal component; **B** representative quality plot, the representative quality of each variable to each principal component; **C** PCA biplot, showing the correlation between the principal component scores of the sample points and the principal components; CK:control environment; DS: drought environment

To understand the difference between the drought and control environments, a principal component analysis was performed (Fig. 3). The scree plot (Fig. 3A) shows that the variance contribution rates of the five principal components were 52.5, 16.3, 13.5, 12.8, and 5%, respectively. The indicator representative quality plot (Fig. 3B) shows that the key information on growth traits (RH, RD) was in the first principal component, and the key information on LS and LRW was in the first, second, and third principal components. The key information for SLA was on the first and fourth principal components. The first and second principal components were extracted to plot the sample scatter plot (Fig. 3C). There were significant differences in the five drought-related traits of the F_1_ population under drought and in the control environment, which also indicates that these five traits can be used as evaluation indicators of drought resistance.

### QTL mapping for drought-related traits

**Fig. 4:**
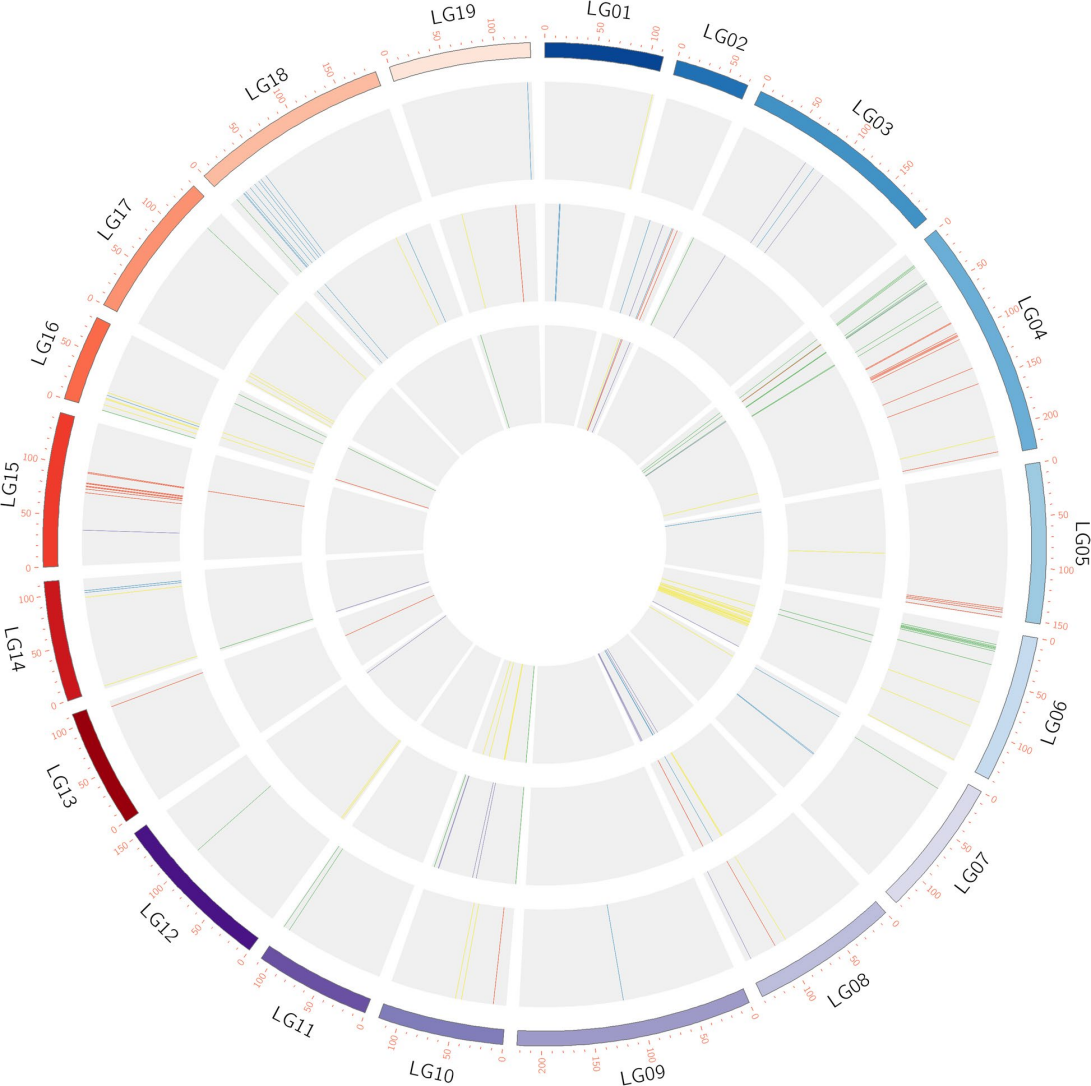
Circle map of the linkage group positions of QTLs for drought-related traits. The first circle represents 19 linkage groups, scale for cM; the 2nd, 3rd and 4th circles are the positions of the QTLs in the 19 linkage groups under the control environment, drought environment, and drought tolerance index, respectively. The relative height growth, relative diameter growth, leaf senescence, specific leaf area and leaf relative water content QTLs are represented by red, blue, yellow, purple and green lines, respectively

A high-density genetic linkage map, including 5796 SNPs for 500 genotypes through whole-genome resequencing, was used to detect the QTLs. The QTL mapping results showed that 92, 63 and 53 QTLs were localized for five traits under the control and drought stress and drought index conditions, respectively, and they were distributed over 19 linkage groups (Table 2, Fig. 4). The number of QTLs for each drought tolerance trait varied from 3 to 25 under different conditions, the LOD values varied from 3.01 to 5.51, and the explained phenotypic variance ranged from 9.0 to 16.0%. The locus qCDR-LG15-3, with 16% explained phenotypic variation, was the main QTL, and all other the QTLs were marginally effective. The number of QTLs for drought tolerance traits showed a decreasing trend in the control, drought stress and drought index conditions. Pleiotropism is defined as one or a pair of genes on chromosomes that affect multiple biological traits. There were two pleiotropic QTLs that regulate RH and RD on LG5.

**Table 2:** Summary of QTLs identified for drought tolerance related traits in poplar populations

Traits	Treatment	Linkage group	QTL number	Logarithm of odds	Phenotypic explanation rate (%)
**Relative height growth (RH)**	Control	LG4-5,LG8,LG10,LG13,LG15	25	3.01-4.47	9.1-13.1
	Drought	LG2,LG4,LG8,LG15,LG19	8	3.01-4.96	9.0-14.5
	Drought index	LG2,LG13,LG16	3	3.16-4.08	9.5-12.1
**Relative diameter gowth (RD)**	Control	LG3,LG5,LG9,LG14-16,LG18-19	22	3.02-5.51	9.1-16.0
	Drought	LG1-2,LG7-8,LG18	11	3.07-4.63	9.2-13.6
	Drought index	LG5,LG8	3	3.3-3.95	9.9-11.7
**Leaf senecence (LS)**	Control	LG1,LG4,LG6,LG8,LG10,LG14,LG16	15	3.01-4.02	9.1-11.9
	Drought	LG5,LG8,LG12,LG16-19	15	3.05-3.91	9.2-11.6
	Drought index	LG2,LG4,LG6,LG7,LG10	24	3.0-4.5	9.0-13.2
**Specific leaf area (SLA)**	Control	LG3-4,LG8,LG15	5	3.08-4.03	9.3-11.9
	Drought	LG2-3,LG10	9	3.07-3.43	9.2-10.3
	Drought index	LG2,LG4,LG6,LG8,LG12,LG14	17	3.01-4.52	9.1-13.3
**Leaf relative water content (LRW)**	Control	LG4,LG6,LG7,LG11-12,LG16-18	25	3.02-4.33	9.1-12.8
	Drought	LG3-4,LG6,LG10,LG14,LG16	20	3.03-5.15	9.1-15.0
	Drought index	LG4,LG10,LG16,LG19	6	3.18-3.78	9.5-11.2

The same QTL was present in different environments (Table 2, Table S1); for example, the same QTL for plant height relative growth was observed under control and drought stress environments, in LG15, with 10.6 and 9.4% phenotypic variance explained, respectively. There were two identical QTLs for LS in the control group, the drought stress group and the drought index group, in LG4, LG6, LG8, and LG16, respectively. The SLA trait had the same QTLs in the control group and drought index, which were distributed in LG8, and the explanation rates of phenotypic variation were 11.9 and 12.9%, respectively. The LRW trait had two identical QTLs in the control group and the drought stress group in LG4, and the phenotypic variation explanation rates were 12.8 and 9.2% and 10.0 and 9.4%, respectively. The LRW trait had the same QTLs in the control group and drought index, which were in LG4, and the phenotypic variation explanation rates were 11 and 9.6%, The LRW trait has four common QTLs under the drought stress group and drought index, and the same three QTLs are located on LG4 and LG16. The phenotypic variation interpretation rates were 15, 13, and 10.2% and 10.2, 9.6, and 9.5%. The QTLs (qDLRWC-LG10-1 and qLRWCI-LG10-1) on LG10 correspond to 10.4 and 10.2% of the phenotypic variation, respectively. The molecular marker np2841 associated with this locus comes from the drought tolerance parent ‘Tongliao 1’ poplar and was initially defined as a molecular marker for drought tolerance. The same QTLs for drought resistance-related traits are present under different water conditions, showing that there is an interaction between the genotype and the environment.

### Candidate gene identification

The genetic regulation effect of specific QTLs in a specific environment is stronger than that of QTLs that are stably present in different environments, so the QTL under a drought environment has an important drought tolerance function. A total of 187 candidate genes were mined from 63 QTL regions of drought-related traits under drought conditions (Table S2). Among them, 121 candidate genes had homologous genes in *Arabidopsis*, and 137 candidate genes had functional annotation information on the reference genome of *Populus trichocarpa*. The annotation results showed that genes with different functions were potentially involved in regulating the drought response of poplars, such as the transcription factors GRAS, MYB, and NAC. Under drought conditions, the RH, RD, LS, SLA, and LRW trait-specific QTL regions contained 24, 42, 52, 39, and 58 candidate genes, respectively.

**Fig. 5:**
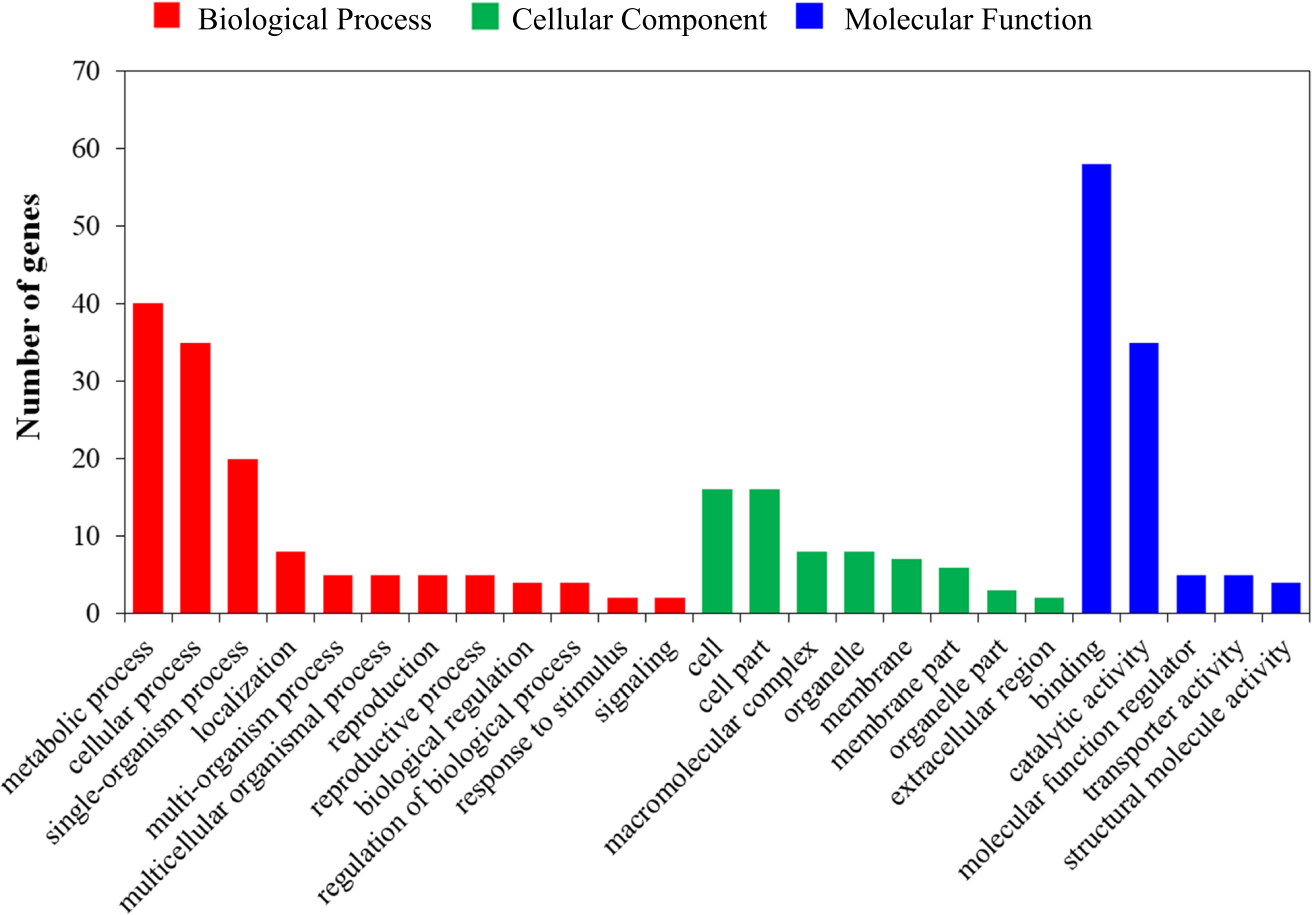
GO enrichment analysis of candidate genes for QTLs of drought tolerance related traits

Two candidate genes, *Potri.003G171300* and *Potri.012G123900*, which are distributed on qDLS-LG18-1 and qDSLA-LG10-4 QTLs encode F-box only protein 6 and Ca^2+^-independent phospholipase A_2_, respectively, and homologues in *Arabidopsis* are involved in genetic regulation in response to drought stress. The GO enrichment analysis of all the candidate genes was divided into three types: cellular component (8 terms), molecular function (5 terms) and biological process (12 terms) (Fig. 5). The key terms included binding, catalytic activity, metabolic process, cellular process, cell, and cell part.

### Candidate gene coexpression network

**Fig. 6:**
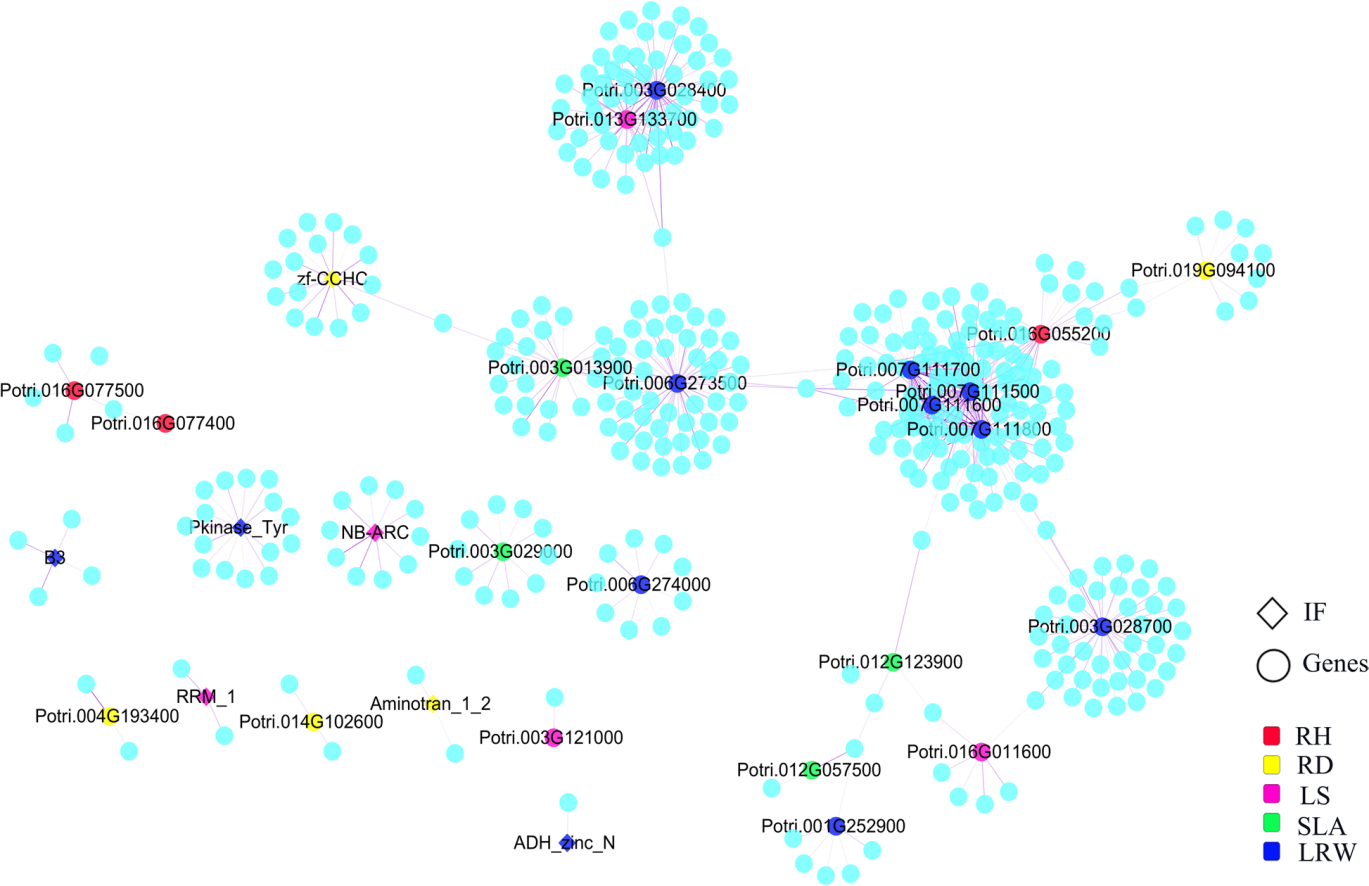
Coexpression network analysis of candidate genes for the drought-related traits QTL. Red: Candidate genes for relative height growth (RH) QTLs; Yellow: Candidate genes for relative diameter growth (RD) QTLs; Pink: Candidate genes for leaf senescence number (LS) QTLs; Green: Candidate genes for specific leaf area (SLA) QTLs; Blue: Candidate genes for leaf relative water content (LRW) QTLs

The poplargene web service is a publicly available gene network resource and network-assisted gene prioritization service that provides the poplar community with a number of useful functions. We downloaded poplar co-expression network data from the database, and selected data related to candidate genes to construct a coexpression network (Fig. 6). Candidate genes for the LRW trait are more closely linked to candidate genes for other traits, which indicates that LRW is the main representative trait for drought tolerance assessment. Candidate genes at key positions in the coexpression network included: *Potri.016G055200*, *Potri.019G094100*, *Potri.004G193500*, *Potri.013G133700*, *Potri.016G011600*, *Potri.003G013900*, *Potri.012G057500*, *Potri.012G123900*, *Potri.003G028400*, *Potri.003G028700*, *Potri.001G252900*, *Potri.006G273500*, *Potri.007G111500*, *Potri.007G111600*, *Potri.007G111700*, and *Potri.007G111800*.

## Discussion

Drought stress usually causes changes in plant morphological, physiological, and biochemical processes [32], which affects plant growth and development, leading to reduced leaf number, and restricts the transport of nutrients to the leaves and the reduces specific leaf area [33]. The relative water content of leaves is used to determine the water status of plants under drought stress, reflecting tissue metabolic activity, and it is the most significant physiological trait for evaluating plant dehydration and drought tolerance [34, 35]. This study showed that the plant height, ground diameter, specific leaf area, and leaf relative water content significantly decreased under drought stress, showing that drought not only inhibited poplar growth and development but also reduced the water holding capacity of leaves, which is consistent with the results of previous studies [7, 36]. Growth traits such as the plant height and ground diameter, leaf traits such as the specific leaf area, leaf relative water content, and leaf senescence number can be used as indicators for drought tolerance evaluation, with leaf relative water content being the most representative.

Drought-related traits such as the plant height and diameter are quantitative characteristics that are regulated by multiple genetic loci, with each locus contributing to weak and cumulative genetic effects. Therefore, resolving the regulatory loci of drought tolerance traits is a prerequisite for breeding for the molecular design underlying drought tolerance. To date, QTL mapping studies have been widely used to resolve the genetic regulatory basis of drought-related traits in crops such as maize, soybean, rice and barley [37–42]. However, there are fewer studies on the genetic regulatory loci for drought-related traits in woody plants [43]. Drought-related traits show different levels of heritability in the F_1_ population, contributing to the mapping of QTLs. This study combined high-density genetic maps and drought-related traits with QTL mapping under different water gradient conditions. Only one QTL, qCDR-LG15-3, which regulates the relative growth of the basal diameter, exceeded 15% of the phenotypic explanation rate, showing that growth-related traits are regulated by a genetic mechanism consisting of both master and micro-effective genes [44, 45]. Bradshaw et al. used poplar F_2_ populations to identify dominant QTLs for regulating growth traits, with a range of 24-33% phenotypic explanation rates [46]. Master effective QTLs controlling growth-related traits were also identified in QTL mapping studies in different plants, such as pine, eucalyptus, oak, and maize [8, 47–50].

In this study, we found that drought tolerance traits were regulated by common and specific QTLs with different genetic effects under two different moisture conditions, and the genetic regulation intensity was higher under drought stress conditions than in the control group. This QTL genetic regulation pattern was also present in different crops, such as maize and barley [39, 42, 51–53]. Under different water gradient conditions, the number of common QTLs for drought-related traits varied from 0 to 7, showing that there was a reciprocal effect between genotypes and different water gradients for this trait, resulting in differences in genetic regulation mechanisms. The five drought-related traits had the same QTL and different QTLs in different treatment environments, showing that there was an interaction effect between the genotype and the environment [54]. QTLs of drought-related traits in drought environments can better regulate the genetic mechanism of drought tolerance in plants [54]. We identified two genes (*Potri.003G171300* and *Potri.012G123900*) that are potentially involved in the drought stress response in QTL regions for drought-related traits under a drought environment [55–57]. *Potri.003G171300* encodes the F-box protein, and its homologous gene (*At1g27340*) in *A. thaliana* negatively regulates the drought stress response by binding to mRNA394 [56]. In addition, F-box family proteins have been reported to be involved in regulating different abiotic stresses; for example, overexpression of the F-box gene (*Os02g44990*) in rice leads to reduced resistance to abiotic stress and enhanced root growth and development [58, 59]. *Potri.012G123900* encodes a Ca^2+^-independent phospholipase A2, and its homologous gene (*At3g54950*) in *A. thaliana* significantly upregulates expression under drought stress and enhances drought tolerance by inhibiting cell membrane lipid degradation [57, 60]. The tropical plant cowpea also contained the fat trophic protein gene VuPAT1 was significantly up-regulated in response to drought stress [61]. These candidate genes provide new gene resources to support poplar transgenic breeding for drought tolerance.

The drought index is used to measure the drought tolerance of a plant. The drought tolerance index of 5 traits was used to divide the hybrid populations into 5 different drought tolerance types and then helped us screen excellent individuals for drought tolerance. The results showed that the QTLs for each drought-related trait in the drought index group accounted for 8-55% of all groups and that partially identical QTLs were present, suggesting that the poplar response to drought stress is subject to complex genetic regulation. Frova et al. found common and specific QTLs for yield traits in maize under different water gradients and drought indices, showing the complexity and specificity of the genetic regulatory mechanisms underlying plant responses to drought stress and drought tolerance [51, 62, 63]. In conclusion, to develop molecular markers for the screening of drought tolerance materials, QTL-linked markers that are stable in different environments and have a large explanation rate of phenotypic variation should be selected. These QTL-linked markers are highly genetically regulated, which improves the probability of beneficial selection of breeding materials [62, 64]. The leaf relative water content is the most meaningful physiological indicator for evaluating the drought tolerance of plant dehydration. A common QTL (qDLRWC-LG10-1) regulating this trait, with phenotypic variance explained at 10.4 and 9.6% under drought stress and drought index, respectively, met the conditions for the screening of markers associated with the target trait. The allele of the marker np2841 associated with this locus came from a drought tolerant parent (‘Tongliao 1’) and was tentatively considered an ideal candidate marker for screening drought-tolerant poplar material.

The regulation of biological processes involves a network of various genes that function in a complex and coordinated manner. However, to date, most studies on *Populus* have been focused on a single or a limited number of genes [65–68]. Functional gene interaction networks are a powerful tool for functional linkage studies of genes in many organisms, including animals, plants and prokaryotes. Once a comprehensive functional association network is generated, genes for which the function is unknown can be easily annotated based on their association with genes of known function. In addition, network-guided screens can be performed to identify novel candidate genes associated with specific traits [69]. We used the data from the public poplar functional gene network database to construct a coexpression network of candidate genes. Candidate genes for drought-related traits were included in the coexpression network, and we found that the candidate genes for five drought-related traits were closely related and that the candidate genes for leaf water content were in key positions. The *Potri.012G123900* gene is also at a key position in the coexpression network, again verifying the previous results. We obtained five key regulatory genes (*Potri.006G273500*, *Potri.007G111500*, *Potri.007G111600*, *Potri.007G111700*, and *Potri.007G111800*) using coexpression network analysis. Potri.006G273500 encodes a cotton fibre expressed protein. *Potri.007G111500* encodes a trypsin and protease inhibitor. *Potri.007G111600* encodes trypsin and protease inhibitor. *Potri.007G111700* encodes a protein similar to the truncated Kunitz trypsin inhibitor. *Potri.007G111800* encodes a trypsin and protease inhibitor.

## Conclusion

In this study, fast-growing and drought-tolerant F_1_ populations were constructed through cross-breeding, and drought stress tests were conducted on parents and hybrid populations. We determined the drought-related traits, and the results showed that drought stress reduced the plant height relative growth, ground diameter relative growth, specific leaf area and leaf relative water content and increased the number of leaf drops. Through genetic mapping analysis, 208 QTLs were identified, revealing 92, 63 and 53 QTLs under control, drought stress and drought index conditions, respectively. A preliminary molecular marker (np2841) for drought tolerance associated with the leaf relative water content QTL (qDLRWC-LG10-1) was developed. A total of 187 candidate genes were identified from specific QTLs under drought conditions. Two candidate genes, *Potri.003G171300* and *Potri.012G123900*, were found to have potential functions in response to drought stress using the reference genome annotation of *Populus trichocarpa* and the homologous gene analysis of *Arabidopsis*. Five key regulatory genes for the drought response were identified using coexpression network, such as *Potri.006G273500*, *Potri.007G111500*, *Potri.007G111600*, *Potri.007G111700*, and *Potri.007G111800*. This work not only provided candidate molecular markers for the screening of drought tolerant poplar materials but also unearthed new genetic resources for drought tolerance breeding.

## Methods

### Plant material and experimental treatment

The F_1_ populations with *P. deltoides* ‘Danhong’ poplar as the female and *P. simonii* ‘Tongliao 1’ poplar as the male were constructed by artificial controlled pollination (using plant material from the Research Institute of Forestry, Chinese Academy of Forestry, we have ownership). The test materials were the parents and 144 F_1_ populations. All the plants were grown in the Experimental Greenhouse at the Chinese Academy of Forestry.

The cuttings were propagated by selecting uniformly growing branches and planted in 15 × 30 cm pots with a substrate of grass charcoal, vermiculite and carbendazim (10:1:1). Experimental treatments were applied after 2 months of growth. A control environment and a drought environment were set up, with the control (CK): 75-80% soil water content and drought stress (DS): 35-40% relative soil water content, relative soil water content = (soil mass water content/ field water holding capacity × 100%). The control and drought stress groups were replicated three times, with four plants in each replicate, in a randomized group design. The soil was watered thoroughly before the stress treatment to keep the soil water content in each pot consistent, and the water was naturally depleted to the soil stress water gradient after irrigation was stopped. The water was replenished by alternate-day weighing method at 17:00 every day during the experiment to maintain the relative soil water content within the set range, and the stress treatment lasted for 30 days.

### Genetic linkage map construction

The number of SNP markers was counted, and the polymorphic markers between parents were classified into eight segregation types (ab × cd, ef × eg, hk × hk, lm × ll, nn × np, aa × bb, ab × cc and cc × ab). Three marker types (lm × ll, nn × np, and hk × hk) in which one or both parents were heterozygous were selected for genetic mapping based on the highly heterozygous biology of the forest trees using a proposed mapping strategy.

The genetic map of this study population was constructed in advance [9], and it included the parents and 500 F_1_ populations. The genetic map consisted of 5796 SNP markers distributed on 19 linkage groups, with a total genetic distance of 2683.80 cM. The average spacing between markers was 0.46 cM, with a range of 0.15-0.81 cM.

### Investigation of phenotypic traits

We investigated the relative growth of plant height, the relative growth of ground diameter, and the leaf senescence number during the drought treatment (30 days). The leaf area for the seventh leaf of poplar seedling was determined after the drought treatment using a leaf area metre LI-3050C (LI-COR, USA). The fresh weight, saturated fresh weight (soaked in distilled water for 24 hours), and dry weight of the seventh leaf of each poplar seedling were weighed using an electronic balance. The leaf relative water content (LRW) = (leaf fresh weight - leaf dry weight)/ (leaf saturated fresh weight - leaf dry weight) × 100%; specific leaf area (SLA) = leaf area/leaf dry weight; and drought index = drought stress phenotypic trait/control phenotypic trait× 100%. Each trait of every line was measured in four plants and measurements were repeated three times, including the control and drought stress groups.

### Data analysis

The mean, variance, standard deviation, coefficient of variation, and heritability of phenotypic data were calculated using SPSS 21.0 software (IBM, USA) [70, 71]. The T test is used to analyse the differences in phenotypic traits between parents; *, **, *** represent the significance levels of *P* < 0.05, *P* < 0.01, and *P* < 0.001, respectively. A clustering analysis was performed on phenotype traits using the R package pheatmap. The R packages FactoMineR and factoextra were used for principal component analysis and visualization [72]. The R package GGally was used to calculate the Pearson’s correlation of phenotype traits and to visualize the scatter matrix plot.

### QTL mapping and candidate gene analysis

QTL mapping for phenotype traits was performed using the multiple interval mapping (MIM) model with the MapQTL v. 6.0 software [9, 73]. A logarithm of odds (LOD) threshold of 3.0 was chosen as evidence for the presence of QTLs. QTLs under drought conditions were used to mine candidate genes.

The 20 kb upstream and downstream regions of the LOD peak position in the genome were regarded as target traits related to genetic regulation loci, and the genes located within these genome regions were considered potential candidate genes [9, 74]. The functional annotation of these candidate genes was performed in the *P. trichocarpa* reference genome (https://phytozome-next.jgi.doe.gov/info/Ptrichocarpa_v3_1). Gene Ontology (GO) enrichment tests were performed on the candidate genes. GO annotations were created using Blast2GO [75].

### Coexpression network analysis

To understand the biological processes underlying drought tolerance related traits in poplar, coexpression networks of candidate genes were constructed using the public poplar gene network database (PoplarGene, covering ~ 70% of the 41,335 poplar genes) (http://bioinformatics.caf.ac.cn/PoplarGene) [76]. The coexpression network was visualized using Cytoscape software [77].

## Supplementary Information

**Additional file 1: Table S1.** QTLs of drought-related traits under control, drought stress, and drought index.

**Additional file 2: Table S2.** Functional annotation of candidate genes for QTLs in drought environment.

## Data Availability

The re-sequence data was uploaded to the National Genomics Data Center (https://bigd.big.ac.cn/gsa/) under accession number CRA002178.

## Abbreviations

QTL: Quantitative trait locus
RH: Relative height growth
RD: Relative diameter growth
LS: Leaf senescence number
SLA: Specific leaf area
LRW: Leaf relative water content
